# Upregulation of cancer-associated gene expression in activated fibroblasts in a mouse model of non-alcoholic steatohepatitis

**DOI:** 10.1038/s41598-019-56039-0

**Published:** 2019-12-20

**Authors:** Masahiro Asakawa, Michiko Itoh, Takayoshi Suganami, Takeru Sakai, Sayaka Kanai, Ibuki Shirakawa, Xunmei Yuan, Tomomi Hatayama, Shu Shimada, Yoshimitsu Akiyama, Katsuhito Fujiu, Yutaka Inagaki, Ichiro Manabe, Shoji Yamaoka, Tetsuya Yamada, Shinji Tanaka, Yoshihiro Ogawa

**Affiliations:** 1 Department of Molecular Endocrinology and Metabolism, Graduate School of Medical and Dental Sciences, Tokyo Medical and Dental University, Tokyo, Japan; 2 Department of Organ Network and Metabolism, Graduate School of Medical and Dental Sciences, Tokyo Medical and Dental University, Tokyo, Japan; 3 Kanagawa Institute of Industrial Science and Technology, Kawasaki, Japan; 4 Department of Molecular Medicine and Metabolism, Research Institute of Environmental Medicine, Nagoya University, Nagoya, Japan; 5 Department of Immunometabolism, Nagoya University Graduate School of Medicine, Nagoya, Japan; 6 Department of Medicine and Bioregulatory Science, Graduate School of Medical Sciences, Kyushu University, Fukuoka, Japan; 7 Department of Molecular Oncology, Graduate School of Medical and Dental Sciences, Tokyo Medical and Dental University, Tokyo, Japan; 8 Department of Advanced Cardiology, Graduate School of Medicine, The University of Tokyo, Tokyo, Japan; 9 Center for Matrix Biology and Medicine, Graduate School of Medicine, Tokai University, Isehara, Japan; 10 Department of Disease Biology and Molecular Medicine, Chiba University Graduate School of Medicine, Chiba, Japan; 11 Department of Molecular Virology, Tokyo Medical and Dental University, Tokyo, Japan; 12 Department of Molecular and Cellular Metabolism, Graduate School of Medical and Dental Sciences, Tokyo Medical and Dental University, Tokyo, Japan; 13 Japan Agency for Medical Research and Development, CREST, Tokyo, Japan

**Keywords:** Metabolic syndrome, Experimental models of disease, Non-alcoholic steatohepatitis

## Abstract

Non-alcoholic steatohepatitis (NASH), characterized by chronic inflammation and fibrosis, is predicted to be the leading cause of cirrhosis and hepatocellular carcinoma (HCC) in the next decade. Although recent evidence suggests the importance of fibrosis as the strongest determinant of HCC development, the molecular mechanisms underlying NASH-induced carcinogenesis still remain unclear. Here we performed RNA sequencing analysis to compare gene expression profiles of activated fibroblasts prepared from two distinct liver fibrosis models: carbon tetrachloride–induced fibrosis as a model without obesity and HCC and genetically obese melanocortin 4 receptor–deficient (MC4R-KO) mice fed Western diet, which develop steatosis, NASH, and eventually HCC. Our data showed that activated fibroblasts exhibited distinct gene expression patterns in each etiology, and that the ‘pathways in cancer’ were selectively upregulated in the activated fibroblasts from MC4R-KO mice. The most upregulated gene in these pathways was fibroblast growth factor 9 (FGF9), which was induced by metabolic stress such as palmitate. FGF9 exerted anti-apoptotic and pro-migratory effects in fibroblasts and hepatoma cells *in vitro* and accelerated tumor growth in a subcutaneous xenograft model. This study reveals upregulation of cancer-associated gene expression in activated fibroblasts in NASH, which would contribute to the progression from NASH to HCC.

## Introduction

Non-alcoholic fatty liver disease (NAFLD) is considered as the hepatic phenotype of metabolic syndrome, and its prevalence is growing in parallel with the global epidemics of obesity and type 2 diabetes^1,2^. The clinical spectrum of NAFLD ranges from simple steatosis to non-alcoholic steatohepatitis (NASH), the latter of which can proceed to cirrhosis and hepatocellular carcinoma (HCC). Besides chronic infection of hepatitis viruses and alcoholic consumption, much attention has been paid to NASH as a leading cause of HCC in the next decade^3,4^. Although a number of studies indicate the importance of fibrosis as the strongest determinant of HCC development^5–7^, it remains unclear how simple steatosis progresses to NASH, and what provides the carcinogenic tissue microenvironment.

Chronic inflammation is a common molecular basis of a variety of chronic diseases, including metabolic syndrome and cancer. During the course of tissue remodeling caused by chronic inflammation, sustained interaction between stromal cells, such as macrophages and fibroblasts, and parenchymal cells leads to tissue dysfunction. Considering substantial evidence that cancer-associated fibroblasts (CAFs) play key roles in tumor progression and metastasis through creating tumor microenvironment^8^, activated fibroblasts in fibrotic tissues would contribute to tumor development. In response to liver injury, hepatic stellate cells (HSCs) transdifferentiate into activated fibroblasts, which are characterized by specific phenotypes including proliferative, contractile, inflammatory, migratory, and enhanced extracellular matrix (ECM) producing capacities^9,10^. HSCs are activated by multiple interactions with other cell types including parenchymal cells (hepatocytes) and non-parenchymal cells (NPCs)^11–13^, suggesting that fibroblasts harbor distinct activation status in each fibrotic pathology. In addition to the previously reported profibrotic factors such as transforming growth factor-β (TGFβ) and platelet-derived growth factor, the study of fibroblasts in NASH may elucidate specific pathways activated in metabolic stress-induced liver fibrosis, and thus gives insights into the molecular mechanisms underlying liver fibrosis-mediated HCC development. However, isolation of fibroblasts from fibrotic livers is often hampered by tissue stiffness, a small population of fibroblasts, and lack of robust cell surface markers^14^.

To date, various animal models of NASH have been proposed using hepatotoxic agents, genetic engineering, and dietary challenges^15^, whereas few of them exhibit both obesity phenotypes and HCC development. In this regard, we previously reported melanocortin 4 receptor–deficient (MC4R-KO) mice as a novel murine model of NASH and HCC^15,16^. MC4R, a 7-transmembrane G protein–coupled receptor, is expressed in the hypothalamic nuclei and regulates food intake and energy expenditure^17^. MC4R-KO mice exhibit hyperphagic obesity, and then sequentially develop simple steatosis, NASH, and HCC on high-fat diet or Western diet (WD)^16^. The histological features and gene expression patterns of livers from MC4R-KO mice were closely similar to those of human NASH and HCC with metabolic risk factors^16,18^. Using this model, we demonstrated that CD11c^+^ resident macrophages would be a novel macrophage subset that accelerates obesity-induced liver fibrosis^19,20^. However, little is known about activated fibroblasts in the model.

In this study, we compared gene expression profiles of activated fibroblasts prepared from two distinct liver fibrosis models: MC4R-KO mice on WD and chemically induced liver fibrosis using carbon tetrachloride (CCl_4_) as a model of fibrosis without obesity and HCC development. RNA sequencing analysis revealed that activated fibroblasts exhibited distinct gene expression patterns in each etiology, and we focused on fibroblast growth factor 9 (FGF9), which belongs to the ‘pathways in cancer’ selectively upregulated in the activated fibroblasts from MC4R-KO mice. FGF9 is a secretory protein of the FGF family, whose expression has been reported in certain types of carcinomas in liver and other organs^21–23^. Our data showed FGF9 exerted anti-apoptotic and pro-migratory effects *in vitro* and it also accelerated tumor growth in a subcutaneous xenograft model. This study demonstrates the pathogenesis-specific activation of fibroblasts, which may contribute to the progression from NASH to HCC.

## Results

### Isolation of activated fibroblasts from distinct liver fibrosis models

**Figure 1:**
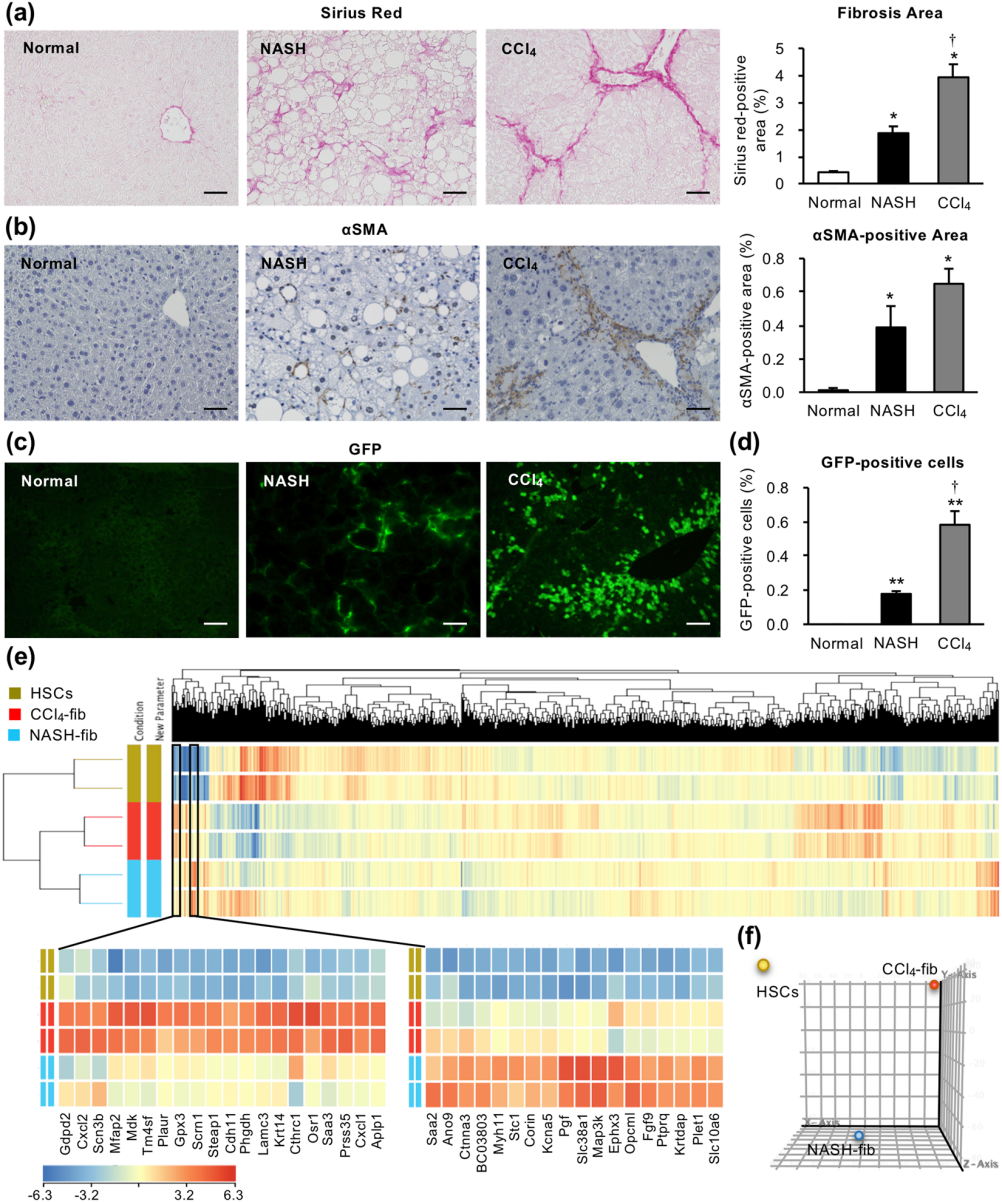
Evaluation of obesity- and carbon tetrachloride-induced liver fibrosis models. Col1a2-GFP Tg mice were crossed with MC4R-KO mice (MC/COL), and were fed Western diet (WD) for 20 weeks to develop NASH (NASH). Col1a2-GFP Tg mice were received carbon tetrachloride (CCl_4_) twice a week for 8 weeks (CCl_4_). (**a**) Sirius red staining and quantification of area positive for Sirius red. (**b**) Activated fibroblasts determined by αSMA immunostaining. (**c**) Type I collagen-producing cells visualized by GFP fluorescence. (**d**) Percentage of fibroblasts (CD45^−^ GFP^+^) in hepatic non-parenchymal cells analyzed by FACS. Scale bars: 50 μm. *n* = 6. *P < 0.05, **P < 0.01 vs. normal. ^†^*P* < 0.05 vs. NASH. Data represent mean ± SEM. (**e**) Unsupervised hierarchical clustering of RNA sequencing datasets of steady-state hepatic stellate cells (HSCs) and activated fibroblasts separated from NASH and CCl_4_-induced fibrotic livers (NASH-fib and CCl_4_-fib, respectively). Lower left and middle panels indicate the gene clusters upregulated selectively in CCl_4_-fib and NASH-fib, respectively. (**f**) Principal component analysis of gene expression data from HSCs, CCl_4_-fib, and NASH-fib.

To visualize activated fibroblasts or collagen-producing cells in the liver, we crossed MC4R-KO mice with collagen α2(I) promoter–driven green fluorescent protein transgenic (Col1a2-GFP Tg) mice (MC/COL mice). MC/COL mice developed NASH-like liver phenotypes after 20-week WD feeding, characterized by steatosis, hepatocellular ballooning, inflammatory cell infiltration, and pericellular fibrosis (Fig. 1a, Supplementary Fig. S1a), as previously reported^16^. We observed pericellular localization of activated fibroblasts or GFP-positive collagen-producing cells (Fig. 1b,c). We also employed a well-established chemically induced fibrosis model to compare the gene expression profiles of activated fibroblasts in both liver fibrosis models with or without HCC development. Col1a2-GFP Tg mice treated with CCl_4_ twice a week for 8 weeks developed perilobular fibrosis (Fig. 1a–c). The fibrosis area and the percentage of GFP-positive fibroblasts in non-parenchymal cells (NPCs) analyzed by flowcytometry were higher in the CCl_4_ model than in the NASH model (Fig. 1a,d, Supplementary Fig. S1b). Since HSCs are the main precursor of activated fibroblasts in the pathogenesis of liver fibrosis^24,25^, we separated HSCs from the wild-type healthy livers by density gradient centrifugation as a normal control. Using these steady-state HSCs and activated fibroblasts from NASH livers (NASH-fib) and CCl_4_-induced fibrotic livers (CCl_4_-fib), we performed RNA sequencing analysis to identify upregulated genes selectively in NASH-fib.

### Gene expression profiles of activated fibroblasts in liver fibrosis models

**Figure 2:**
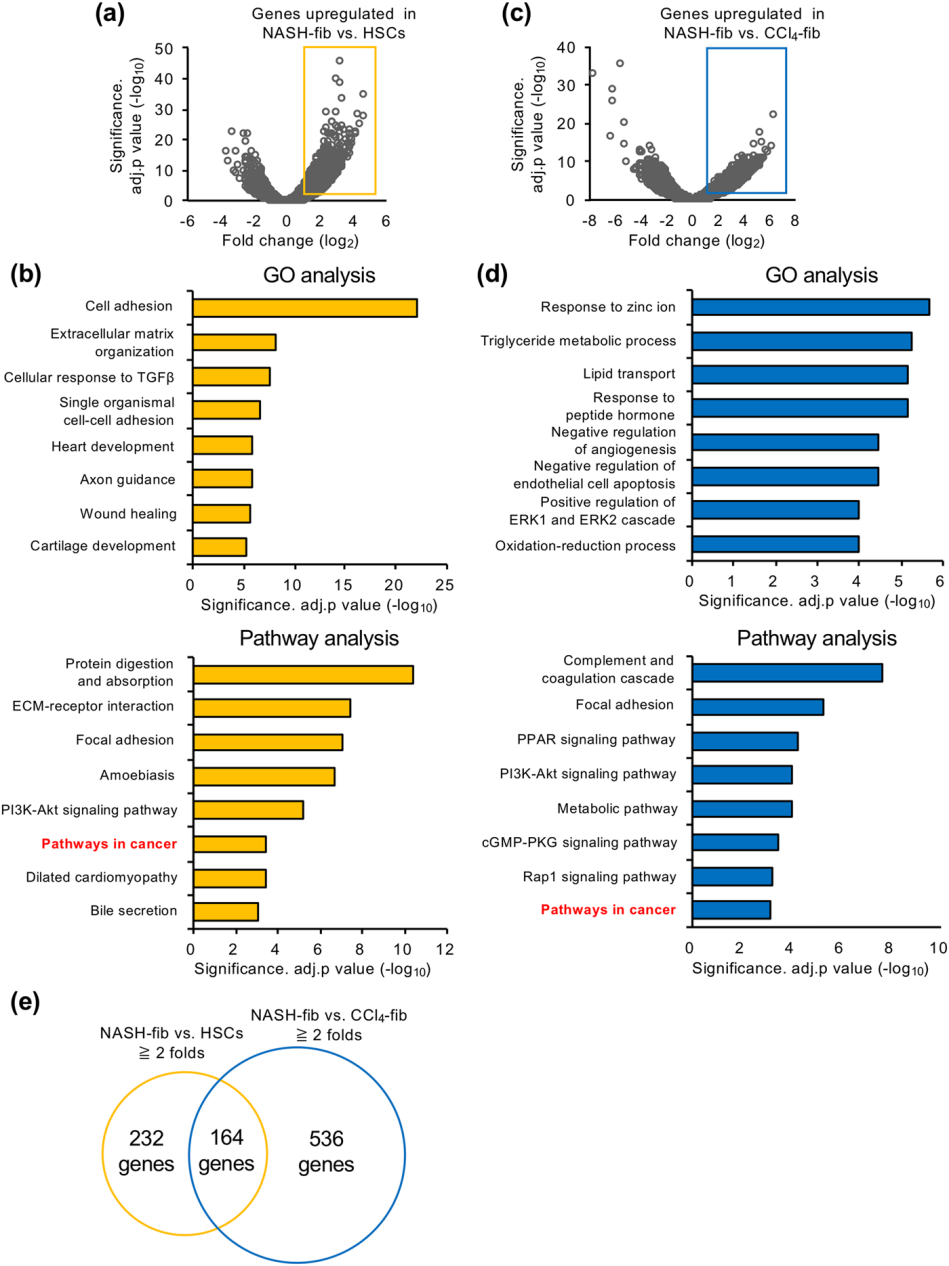
Expression profiling of activated fibroblasts from two distinct liver fibrosis models. (**a**) Volcano plot of RNA sequencing analysis with the comparison between NASH-fib and HSCs. Yellow rectangle denotes significantly upregulated genes in NASH-fib compared with HSCs for more than 2 folds (log_2_ fold change ≧ 1 and adjusted *p* value < 0.05). (**b**) GO and pathway analysis of the 396 genes upregulated in NASH-fib against HSCs. Each top 8 were indicated. (**c**) Volcano plot of RNA sequencing analysis with the comparison between NASH-fib and CCl_4_-fib. Blue rectangle denotes significantly upregulated genes in NASH-fib compared with CCl_4_-fib for more than 2 folds (log_2_ fold change ≧ 1 and adjusted *p* value < 0.05). (**d**) GO and pathway analysis of the 700 genes upregulated in NASH-fib against CCl_4_-fib. (**e**) The overlap between the genes more than 2-fold upregulated (log_2_ fold change ≧ 1) in NASH-fib against HSCs and CCl_4_-fib, respectively.

Unsupervised hierarchical clustering and principle component analysis revealed that activated fibroblasts from different liver fibrosis models showed distinct gene expression patterns (Fig. 1e,f). Interestingly, a gene cluster highly upregulated selectively in CCl_4_-fib included factors already known as their pathophysiological roles in liver injury and fibrosis^26–28^, whereas most of the genes selectively upregulated in NASH-fib haven’t been reported in this context. The comparison between NASH-fib and HSCs was shown in Fig. 2a as a volcano plot. A total of 396 genes were upregulated more than 2-fold (log_2_ fold change ≧ 1) in NASH-fib against HSCs, and GO analysis revealed that genes related to cell adhesion, extracellular matrix organization, cellular response to TGFβ, and wound healing were activated in NASH-fib compared to HSCs (Fig. 2b). On the other hand, GO analysis on 700 genes upregulated more than 2-fold in NASH-fib compared with CCl_4_-fib indicated activation of genes related to lipid metabolism (Fig. 2c,d). Although MC4R-KO mice did not develop liver tumors at this time point, the ‘pathways in cancer’ were activated in NASH-fib compared with HSCs and CCl_4_-fib (Fig. 2b and d). Among 164 genes specific for NASH-fib (Fig. 2e, Supplementary Table S1), we focused on fibroblast growth factor 9 (FGF9), which was the most upregulated gene in the ‘pathways in cancer’. These findings led us to speculate that metabolic stress induces *Fgf9* mRNA expression in activated fibroblasts, thereby contributing to the subsequent tumor development in the NASH model.

### Metabolic stress induces FGF9 expression in activated fibroblasts

**Figure 3:**
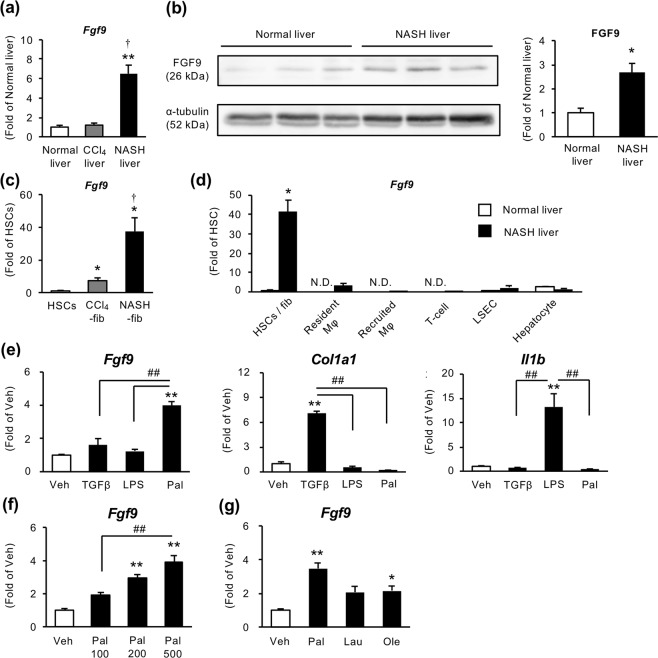
Metabolic stress induces FGF9 expression in NASH-fib. (**a**) Hepatic mRNA expression levels of *Fgf9* in wild-type mice fed standard diet (normal liver), Col1a2-GFP Tg mice received intraperitoneal CCl_4_ injection (CCl_4_ liver), and MC4R-KO mice fed WD for 20 weeks (NASH liver) evaluated by quantitative real-time PCR. *n* = 6. (**b**) Protein expression levels of FGF9 in normal and NASH livers by Western blot analysis. Blots are shown as cropped images. Uncropped Western blot images are included in Supplementary Fig. S2a. *n* = 3. *P < 0.05, **P < 0.01 vs. normal liver; ^†^*P* < 0.05 vs. CCl_4_ liver. (**c**) *Fgf9* mRNA expression levels in isolated HSCs and activated fibroblasts (CCl_4_-Fib, NASH-fib). *n* = 3. (**d**) *Fgf9* mRNA expression levels in various cell types separated from normal and NASH livers. Resident macrophages, CD45^+^ Ly6G^−^ F4/80^hi^ CD11b^lo^; recruited macrophages, CD45^+^ Ly6G^−^ F4/80^lo^ CD11b^hi^; CD4^+^ T cells, CD45^+^ CD4^+^; and liver sinusoidal endothelial cells (LSEC), CD45^−^ CD146^+^. Hepatocytes were isolated from lean wild-type mice and MC4R-KO mice fed WD for 4 weeks. *n* = 3–8. *P < 0.05 vs. HSCs; ^†^*P* < 0.05 vs. CCl_4_-fib. (**e**) mRNA expression levels in cultured HSCs treated with TGFβ (10 ng/ml), lipopolysaccharide (LPS, 10 ng/ml), and palmitic acid (200 μM) for 24 hours. (**f**) Dose-dependent effect of palmitate (100, 200, and 500 μM) on FGF9 induction in HSCs. (**g**) Effect of various fatty acids (200 μM) on FGF9 induction. Lau, laurate; Ole, oleate. *n* = 5. *P < 0.05, **P < 0.01 vs. veh; ^##^*P* < 0.01. Data represent mean ± SEM.

We next examined the expression of FGF9 in the liver. Hepatic *Fgf9* mRNA expression was markedly increased in the liver of MC4R-KO mice fed WD for 20 weeks (NASH liver) compared to normal liver and CCl_4_-induced fibrotic liver (Fig. 3a). FGF9 protein levels were also increased in NASH liver (Fig. 3b, Supplementary Fig. S2a). We confirmed the upregulation of *Fgf9* mRNA expression by qPCR using isolated HSCs and activated fibroblasts (Fig. 3c). To examine cell type–specific expression of FGF9, we isolated hepatocytes, macrophages, T cells, and liver sinusoidal endothelial cells (LSECs) from normal and NASH livers. *Fgf9* mRNA expression was exclusively induced in activated fibroblasts isolated from the NASH liver (Fig. 3d). Upregulation of *Fgf9* was also observed in the livers from wild-type mice fed WD for 32 weeks, at which they develop liver fibrosis (Supplementary Fig. S3a). Interestingly FGF9 mRNA expression was rather downregulated in a methionine and choline–deficient diet (MCDD) model, which is a well-known model of steatohepatitis without obesity and HCC development (Supplementary Fig. S3b). Among already known stimuli involved in the pathogenesis of NASH, we found that palmitate, the most abundant saturated fatty acid in WD-fed animals^29^, significantly induced *Fgf9* mRNA expression in HSCs (Fig. 3e). Immunocytochemistry of HSCs revealed increased FGF9 protein levels after palmitate treatment (Supplementary Fig. S4a,b). We also confirmed the dose-dependent effect of palmitate on FGF9 induction in hepatic stellate cells (HSCs) (Fig. 3f). On the other hand, laurate, another saturated fatty acid, and oleate, a monounsaturated fatty acid, exerted a marginal effect on FGF9 expression (Fig. 3g). These data suggest that metabolic stress such as palmitate stimulate FGF9 expression in liver fibroblasts.

### FGF9 induces inflammatory changes and promotes migration in liver fibroblasts

**Figure 4:**
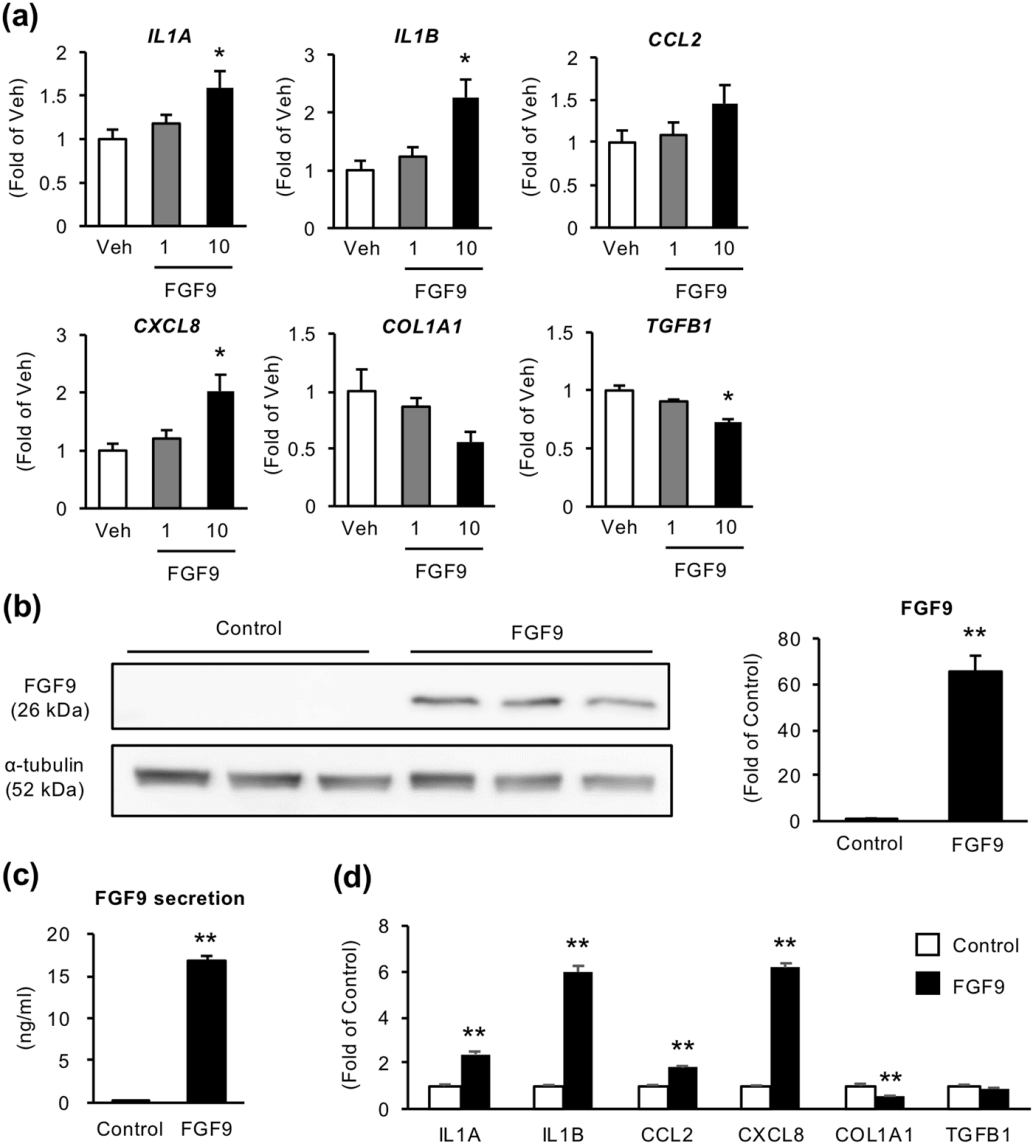
FGF9 induces inflammatory changes in LX2 cells. Human HSC cell line LX2 cells were treated with human recombinant FGF9 at a dose of 1 or 10 ng/ml for 24 hours. (**a**) mRNA expression levels of proinflammatory cytokines (*IL1A*, *IL1B*), chemokines (*CCL2*, *CXCL8*) and fibrogenic factors (*COL1A1*, *TGFB*). *n* = 4. *P < 0.05 vs. veh. FGF9 or GFP (control)-overexpressing LX2 cells (FGF9-LX2 and control-LX2, respectively) were established using lentiviral vectors. Western blot analysis (**b**) and FGF9 secretion (**c**) into culture supernatants using FGF9-overexpressing and control-LX2 cells. Blots are shown as cropped images. Uncropped Western blot images are included in Supplementary Fig. S2b. *n* = 4. **P < 0.01 vs. control-LX2. (**d**) mRNA expression levels of genes related to proinflammatory cytokines, chemokines and fibrogenic factors in FGF9-LX2 cells. *n* = 8. **P < 0.01 vs. control-LX2. Data represent mean ± SEM.

**Figure 5:**
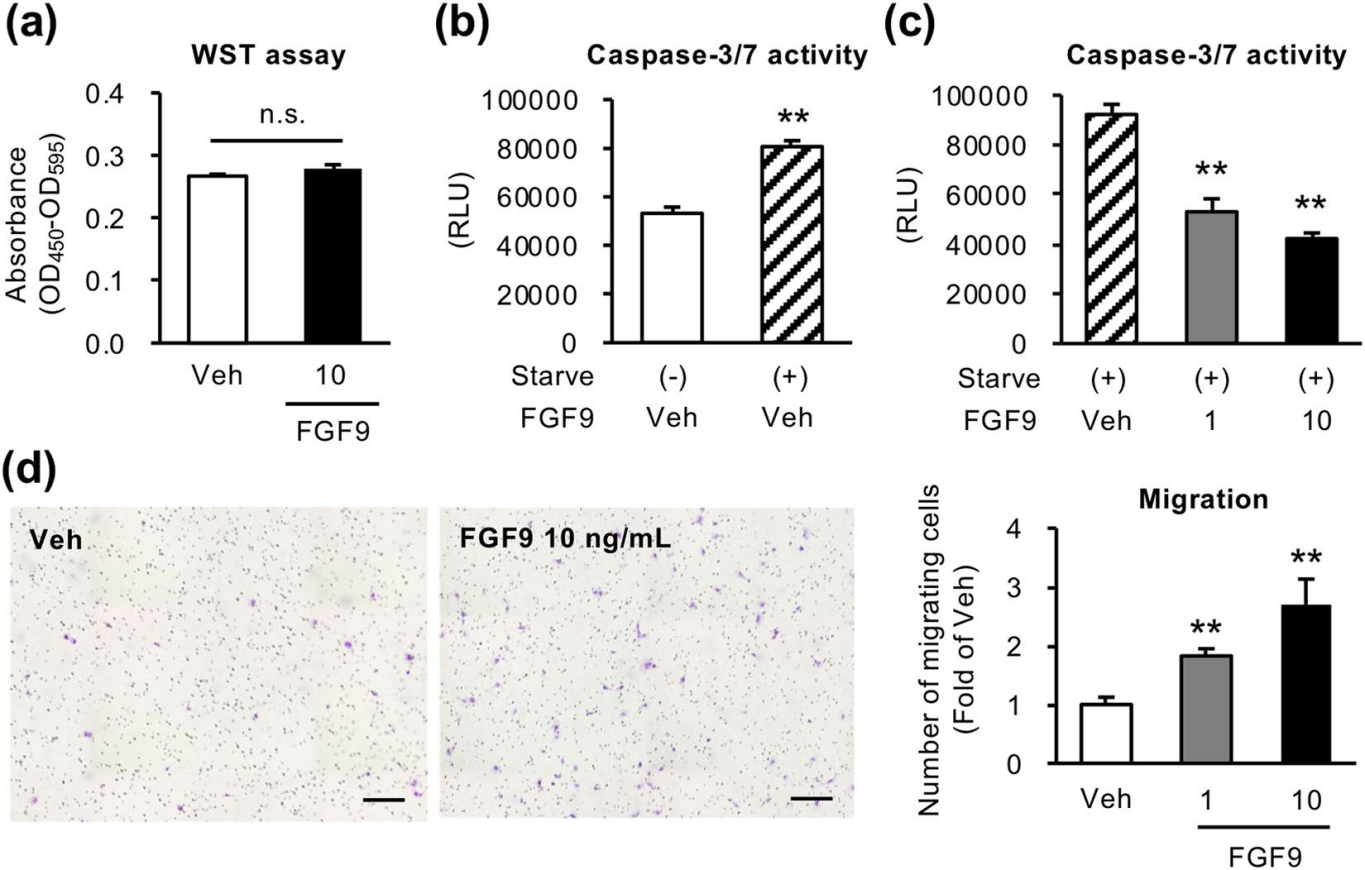
FGF9 enhances cell migration and inhibits apoptosis in LX2 cells. (**a**) Effect of FGF9 on LX2 cell proliferation determined by WST assay after 96-hour incubation. *n* = 8. Effect of serum starvation (starve) for 48 hours (**b**) and coexistence with recombinant FGF9 (c) evaluated by caspase-3/7 activity assay in LX2 cells. *n* = 6. (**d**) Migration activity of FGF9-treated LX2 cells determined by transwell migration assay. LX2 cells were seeded onto the insert of transwell in serum free medium containing recombinant FGF9 (1 or 10 ng/ml), and incubated with medium containing 2% FBS in the lower chamber for 24 hours. *n* = 4. **P < 0.01 vs. starve (−) or veh. n.s., not significant. Data represent mean ± SEM.

Next, we examined the effect of recombinant human FGF9 on activation of fibroblasts using human HSC cell line LX2 cells. FGF9 treatment markedly increased mRNA expression of proinflammatory cytokines and chemokines (interleukin 1α (*IL1A*), interleukin 1β (*IL1B*), C-C chemokine ligand 2 (*CCL2*) and C-X-C motif ligand chemokine 8 (*CXCL8*)) (Fig. 4a). On the other hand, expression of profibrogenic genes (collagen type α1(I) (*COL1A1*) and *TGFB*) was not changed or rather decreased by FGF9 treatment (Fig. 4a). Using stably FGF9-overexpressing LX2 cells (FGF9-LX2 cells) (Fig. 4b,c, Supplementary Fig. S2b), we confirmed the consistent results with LX2 cells treated with recombinant FGF9 (Fig. 4d). Moreover, we examined the effect of FGF9 on cell apoptosis, proliferation, and migration. Although it is known that FGF9 exerts a mitogenic effect on certain cell types^30^, WST assay detected no appreciable effect of recombinant FGF9 on proliferation in LX2 cells (Fig. 5a). On the other hand, FGF9 treatment significantly suppressed serum starvation–induced apoptosis and increased cell migration ability of LX2 cells (Fig. 5b–d). Cell migration was also decreased when FGF9 expression was suppressed by siRNA in FGF9-LX2 (Supplementary Fig. S5a–c). These data suggest that FGF9 elicits proinflammatory phenotypes and affects cellular dynamics in activated fibroblasts, which may contribute to liver fibrosis and HCC development.

### FGF9 promotes the growth of liver tumor *in vivo*

**Figure 6:**
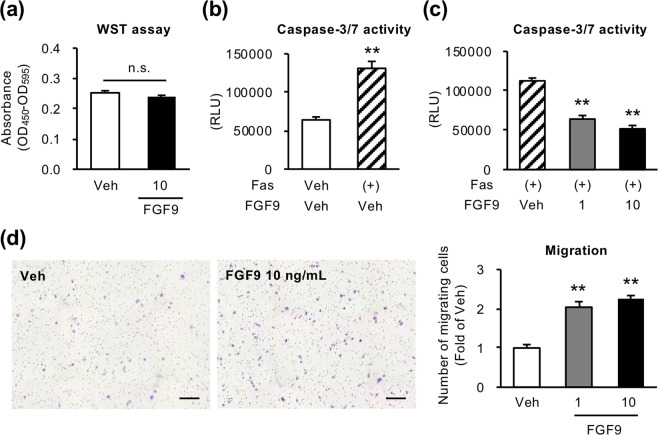
FGF9 enhances cell migration and inhibits apoptosis in HepG2 cells. (**a**) Effect of FGF9 on HepG2 cell proliferation determined by WST assay after 96-hour incubation. *n* = 8. Effect of anti-Fas antibody (Fas) at a dose of 100 ng/ml for 24 hours (**b**) and coexistence with recombinant FGF9 (**c**) evaluated by caspase-3/7 activity assay in HepG2 cells. *n* = 6. (**d**) Migration activity of FGF9-treated HepG2 cells determined by transwell migration assay. HepG2 cells were seeded onto the insert of transwell in serum free medium containing recombinant FGF9 (1 or 10 ng/ml), and incubated with medium containing 2% FBS in the lower chamber for 24 hours. *n* = 4. **P < 0.01 vs. veh. n.s., not significant. Data represent mean ± SEM.

**Figure 7:**
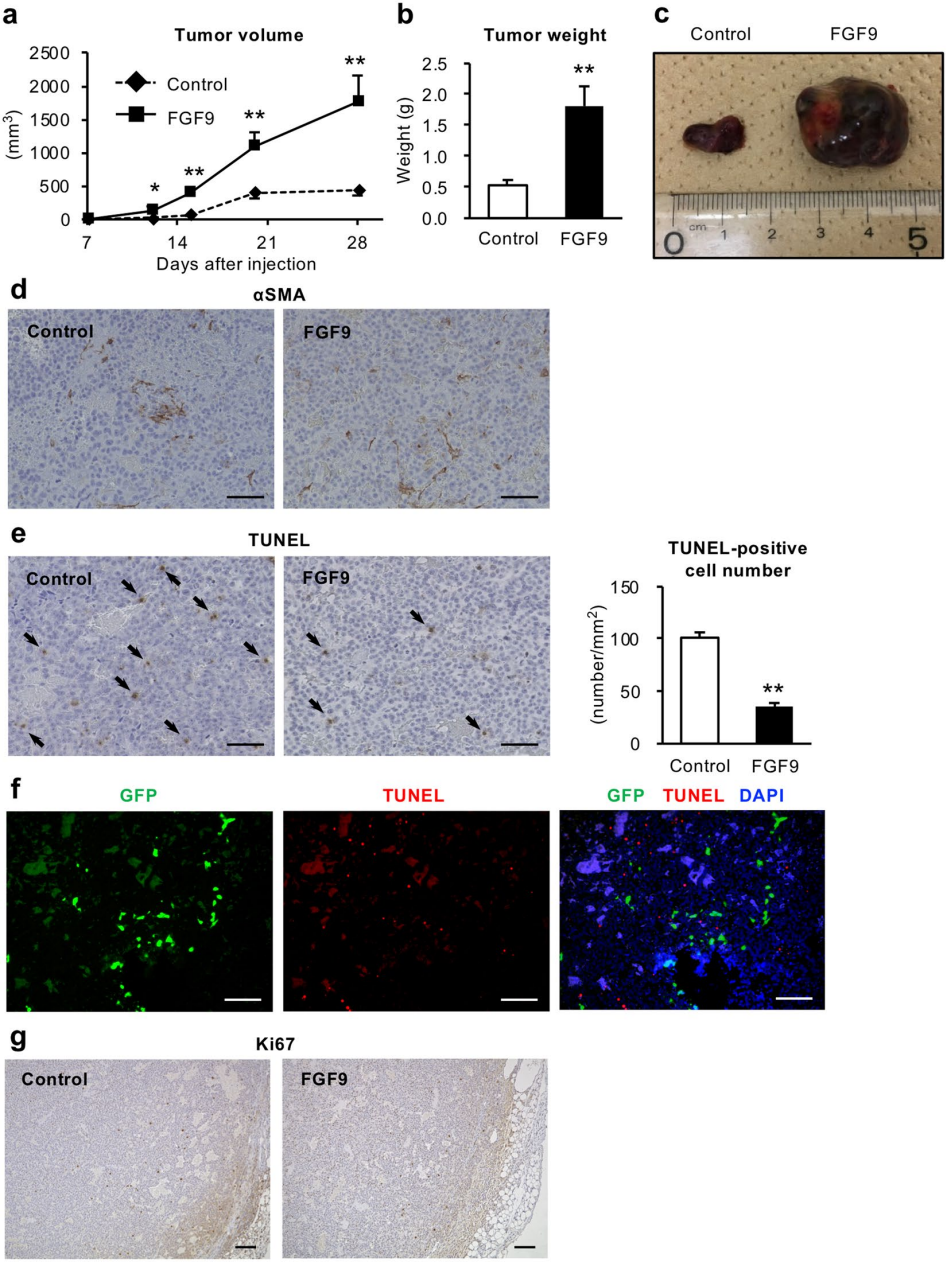
FGF9 promotes tumor growth in a human tumor xenograft model. HepG2 cells (2 × 10^5^ cells) together with control-LX2 or FGF9-LX2 cells (1 × 10^6^ cells) were transplanted subcutaneously in the flank of immunodeficient mice. (**a**) Time course of the tumor volume. Weight (**b**) and representative images (**c**) of subcutaneous tumors at 4 weeks after transplantation. αSMA staining (**d**) and TUNEL staining (**e**) of the tumors. Arrows indicate TUNEL-positive cells. (**f**) GFP and TUNEL double immunofluorescent staining of the tumors that includes HepG2 cells and control (GFP)-LX2 cells. (**g**) Ki67 immunostaining of the tumors. Scale bars: 100 μm. *P < 0.05, **P < 0.01 vs. control. *n* = 7. Data represent mean ± SEM.

We next examined the effect of FGF9 on hepatoma cell line HepG2 cells. Similar to the effect on LX2 cells, FGF9 treatment did not change cell proliferation of HepG2 cells, but significantly suppressed anti-Fas antibody-induced apoptosis and increased cell migration ability (Fig. 6). We further examined the effect of FGF9 on tumor growth *in vivo* using a human tumor xenograft model (Fig. 7). HepG2 cells were subcutaneously xenografted in immunodeficient mice together with control-LX2 cells or FGF9-LX2 cells. The tumor volume as well as the tumor weight was remarkably increased when HepG2 cells were co-transplanted with FGF9-LX2 cells relative to control-LX2 cells (Fig. 7a–c). Histological analysis revealed that tumors comprise mainly HepG2 cells, and that both control- and FGF9-LX2 cells were dispersed in the tumors (Fig. 7d). Co-transplantation of HepG2 cells with FGF9-LX2 cells significantly reduced the number of TUNEL-positive cells relative to that with control-LX2 cells (Fig. 7e). We performed GFP and TUNEL double immunofluorescent staining using the subcutaneous tumor that includes HepG2 cells, and found that most of the TUNEL-positive cells were negative for GFP, suggesting that apoptotic cells are mainly HepG2 cells (Fig. 7f). Ki67 immunostaining revealed a similar distribution pattern of Ki67-positive cells in the marginal zone of tumors in both groups transplanted with control-LX2 and FGF9-LX2 cells (Fig. 7g). These observations suggest that liver fibroblast-derived FGF9 promotes the growth of liver tumor *in vivo*, at least partly, through suppression of cancer cell apoptosis.

## Discussion

Fibroblasts in normal tissues are generally inert with low levels of secretory and synthetic properties to maintain the structural framework and tissue homeostasis. In response to injury and stress in parenchymal cells, fibroblasts are activated to produce ECM molecules and various cytokines accompanied by enhanced migratory and contractile functions. In this study, we demonstrate for the first time that activated fibroblasts in a murine model of NASH show unique gene expression profiles, which would be involved in tumorigenesis during the progression from NASH to HCC. Although evidence has accumulated that fibroblasts are highly heterogeneous, either quiescent or activated, depending on each organ and pathogenic etiology^31,32^, the functional diversity is far well understood, at least partly due to lack of specific markers for fibroblasts and appropriate animal models to investigate the pathophysiologic role of fibroblasts. In this regard, we employed Col1a2-GFP Tg mice, which enable us to separate activated fibroblasts based on the capacity of collagen production^24^. Moreover, MC4R-KO mice fed WD sequentially develop simple steatosis, liver fibrosis, and finally HCC as a result of obesity-induced systemic metabolic derangements, since MC4R expression is undetectable in the liver^16^. Using this model and chemically induced liver fibrosis model, we found distinct gene expression patterns of activated fibroblasts. It is noteworthy that the ‘pathways in cancer’ were upregulated in activated fibroblasts from livers of MC4R-KO mice fed WD for 20 week, at which they exhibited NASH-like liver phenotypes without HCC development. Therefore, this study indicates that activated fibroblasts in NASH exhibit, in addition to the proinflammatory properties, tumor-promoting phenotypes.

FGF9 is a secretory protein of the FGF family, and activates paracrine or autocrine signaling via FGF receptors^33–35^. FGF9 signaling regulates a variety of cell biological behaviors, including proliferation, differentiation, survival, and motility in the embryogenesis and the physiological status^36^. For instance, FGF9 promotes cell proliferation of hepatocytes *in vitro*^37^. It is also reported that FGF9 is expressed in poor-prognostic carcinoma cells in various organs including liver^21–23,38^, and CAFs in gastric carcinoma^39^. However, the pathogenic role of FGF9 in the progression from NASH to HCC remains to be elucidated. In this study, we demonstrated that FGF9 exerts proinflammatory, pro-migratory, and anti-apoptotic effects, rather than activates fibrogenic pathways in fibroblasts. Cytokines and chemokines upregulated by FGF9 treatment are known to play important roles in activation of neutrophils, macrophages, regulatory T cells, and endothelial cells, contributing to liver fibrosis and tumor development^40,41^. Although various immune cells also produce these cytokines and chemokines, FGF9-producing fibroblasts may constitute a microenvironment that facilitates hepatic fibrosis and tumor growth through induction of local inflammation. When HSCs undergo transdifferentiation into activated fibroblasts after liver injury or stress, they obtain proliferative and migratory capacities, thereby inducing liver fibrosis^9^. Taken together, we speculate that FGF9 contributes to the development of NASH/HCC through alterations in cellular dynamics and immunomodulatory effects.

It is also important to know how FGF9 expression is induced in fibroblasts from NASH livers. To date, limited information has been provided about the regulatory mechanism of FGF9 expression, such as prostaglandin E2 and hypoxia in endometriotic stromal cells and colon cancer cells, respectively^42,43^. This study provides evidence that palmitate, a typical metabolic stress in NAFLD/NASH, induces FGF9 expression in primary HSCs. It has been reported that palmitate exerts its effects through multiple mechanisms: binding to cell surface receptors, increased production of proinflammatory metabolites, and impaired function of cellular organelles. Since LPS didn’t have an appreciable impact on FGF9 expression in primary HSCs, it is not likely that TLR4 signaling is involved in the mechanism. Our preliminary data showed that U0126, an ERK1/2 inhibitor, significantly suppressed the palmitate-induced FGF9 expression in HSCs (Itoh *et al*. unpublished data), suggesting that palmitate upregulates FGF9 expression, at least partly, through activation of ERK signaling. Although further studies are required to fully understand the regulatory mechanisms of FGF9 expression, our data would help understand the NASH-specific activation status of fibroblasts and its pathogenic role in the development of HCC.

It is now widely accepted that cancer progression and metastasis are not solely dependent on cancer cell-autonomous defects, but are controlled by the tumor microenvironment^8^. Indeed, substantial attention has been paid to CAFs and tumor-associated macrophages (TAMs). In this regard, FGF9 is exclusively upregulated in fibroblasts in the liver, suggesting its paracrine effect on tumor growth. On the other hand, fibrosis-associated fibroblasts and CAFs share similar cell biology, such as migration, secretory phenotypes, and ECM production, and thus the functional differences between these fibroblasts are yet to be clearly defined at the molecular level. In addition, recent evidence has pointed to the role of senescence-associated secretory phenotype (SASP) in fibroblasts in tumor development and suppression^44^. For instance, Yoshimoto *et al*. reported that dietary obesity alters gut microbiota and increases the levels of deoxycholic acid in the enterohepatic circulation, thereby provoking SASP of HSCs to promote HCC development^45^. In contrast, Lujambio *et al*. reported that p53-dependent senescence programs in HSCs suppress tumorigenesis through the recruitment of anti-tumor M1 macrophages in a murine model using CCl_4_ and diethylnitrosamine^46^. Further studies are required to clarify whether FGF9 is categorized as a factor related to SASP. These discussions are reminiscent of an emerging concept that macrophages are highly heterogenous and composed of multiple subsets with functional diversity. For instance, we and others recently reported that there are unique macrophage subsets specific for the respective etiologies in the development of tissue fibrosis in the liver and lung^19,47^. Similarly, Shook *et al*. pointed out the heterogeneity of fibroblasts, which is supported by a certain macrophage subset in a skin repair model^48^. Given the disease-specific fibroblast subsets have been identified, it would give insights into understanding the molecular mechanisms and developing novel therapeutic strategies of chronic inflammatory diseases including NASH.

It is critically important whether our observations in this study could be generalized in other mouse models of NASH. As shown in Supplementary Fig. S3a, we confirmed FGF9 upregulation in the livers from wildtype mice fed WD for 32 weeks, whereas FGF9 mRNA expression was rather downregulated in the livers from wildtype mice fed MCDD for 8 weeks. Because the MCDD model shows a marked reduction in body weight, these observations suggest that, in addition to the local microenvironment in the liver, systemic metabolic conditions may affect FGF9 expression in HSCs. Moreover the xenograft model in this study was performed only with HepG2 cell line. In the next step, we need to elucidate the role of FGF9 in the development of NASH/HCC using other cell lines and/or other animal models. Fibroblast-specific FGF9–deficient mice would be useful to investigate the causal relationships between fibroblast-derived FGF9 and disease development. In addition, it is interesting to verify the clinical implication of our findings; whether FGF9 is upregulated in human NASH, but not in liver fibrosis due to other etiologies.

In summary, we demonstrate that activated fibroblasts from metabolic stress-induced liver fibrosis exhibit distinct gene expression patterns compared to those from chemically induced liver fibrosis. To the best of our knowledge, this study reveals for the first time upregulation of cancer-associated gene expression in activated fibroblasts in NASH. Our data also show that metabolic stress such as palmitate induces FGF9 expression in activated fibroblasts, thereby increasing cell migration and viability in fibroblasts and hepatoma cells *in vitro*, and thus promoting tumor growth *in vivo* (Supplementary Fig. 6). This study would give insights into pathogenesis-specific activation status of fibroblasts, and the underlying mechanisms of the development of HCC from NASH.

## Materials and Methods

### Reagent

All reagents were purchased from Sigma-Aldrich (St Louis, MO, USA) or Nacalai Tesque (Kyoto, Japan) unless otherwise noted.

### Animals

MC4R-KO mice on the C57BL/6J background were kindly provided by Joel K. Elmquist (University of Texas Southwestern Medical Center, Dallas, Texas, USA)^17^, and Col1a2-GFP Tg mice were previously described^24^. C57BL/6 J wild-type mice and NOD/ShiJic-*scid*Jcl mice were purchased from CLEA Japan. MC4R-KO mice and Col1a2-GFP Tg mice were crossed to generate Col1a2-GFP Tg MC4R-KO mice (MC/COL mice). In diet-induced NASH model, 8 week-old male MC/COL mice were fed WD (D12079B; 468 kcal/100 g, 41% energy as fat, 34.0% sucrose, 0.21% cholesterol; Research Diets) for 20 weeks. In CCl_4_-induced liver fibrosis model, Col1a2-GFP Tg mice received intraperitoneal CCl_4_ injection twice a week for 8 weeks at a dose of 1 ml/kg diluted 1:4 in olive oil. MCDD (Dyets, Bethlehem, PA, USA) was used to induce steatohepatitis. At the end of the experiment, they were sacrificed under anesthesia, when fed ad libitum.

### Histological analysis

The liver was fixed with neutral-buffered formalin and embedded in paraffin. Four-μm-thick sections of the liver were stained with Sirius red or α-smooth muscle actin (αSMA) (ab5694, Abcam, Cambridge, UK), and positive areas for Sirius red or αSMA were measured using WinROOF software (Mitani, Tokyo, Japan)^16^. To observe GFP-positive cells, the liver was fixed with 4% paraformaldehyde and embedded in OCT compound and frozen in dry ice acetone. Ten-μm-thick frozen sections were mounted in Vectashield mounting medium with DAPI (Vector Labs, Burlingame, CA, USA) and photographed using BZ-9000 fluorescent microscope (Keyence, Osaka, Japan). For the xenograft model, the subcutaneous tumor was fixed with neutral-buffered formalin and embedded in paraffin. Four-μm-thick sections of the subcutaneous tumor were stained with antibodies against αSMA, GFP (A-11122, Invitrogen, Carlsbad, CA, USA) and Ki67 (ab15580, Abcam, Cambridge, UK). In the immunofluorescent staining for GFP, the fluorescence was amplified using fluorescein tyramide reagent and amplification diluent (PerkinElmer, Waltham, MA, USA). Apoptotic cells were detected by TdT mediated dUTP-biotin nick end labeling (TUNEL) assay using the ApopTag Plus Peroxidase *In Situ* Apoptosis Detection Kit or ApopTag Red *In Situ* Apoptosis Detection Kit (Merck, Darmstadt, Germany), and the numbers of TUNEL-positive cells were counted in the whole area of each section and expressed as the mean number/mm^2,20^.

### Isolation of quiescent HSCs

HSCs were isolated by density gradient-based separation method^14^. In brief, the livers were perfused with Hank’s balanced salt solution (HBSS) without calcium and magnesium from the portal vein to remove blood from the liver, and subsequently dispersed in HBSS with calcium and magnesium supplemented with 1 mg/mL type IV collagenase (Merck, Darmstadt, Germany), 50 μg/mL DNase I (Roche, Basel, Switzerland)^16^. After filtered through a 100 μm cell strainer, the cell suspensions were centrifuged at 50 × g for 3 minutes at 4 °C to remove hepatocytes, and then the supernatants were centrifuged at 800 × g for 5 minutes at 4 °C. The precipitates were resuspended in 6% OptiPrep to make density gradient, and cells in the middle layer were collected. Purity > 90% was confirmed by their shape and autofluorescence excited by UV light (Supplementary Fig. S4a).

### Isolation of cells that constitute the livers

After the livers were digested with type IV collagenase and DNase I, the cell suspensions were centrifuged at 50 × g for 3 minutes to remove hepatocytes. The supernatants were collected by centrifugation at 570 × g for 5 minutes, and resuspended in 30% percoll. The cell suspensions were centrifuged at 570 × g for 15 minutes at room temperature to remove cell debris. Red blood cells were lysed by ACK buffer and nonparenchymal cells were washed twice with PBS and resuspended in PBS containing EDTA and 0.5% BSA. Cells were stained with anti-mouse CD45 antibody (30-F11) and 7-AAD (Biolegend, San Diego, CA, USA) and 7-AAD^−^ CD45^−^ GFP^+^ cells (collagen-producing cells, fibroblasts) were sorted using FACSAriaII (BD Biosciences, San Jose, CA, USA). To separate macrophages, T lymphocyte, and sinusoidal cells, nonparenchymal cells were stained with antibodies against CD45, F4/80 (BM8), CD11b (M1/70), CD4 (GK1.5), CD11c (N418), and CD146 (ME-9F1) (Biolegend, San Diego, CA, USA), and sorted using FACSAriaII.

### RNA sequencing analysis

Total RNAs were extracted from pooled HSCs and fibroblasts of 3–5 mice using the PureLink RNA Micro Kit (three samples per each group). Total RNAs were amplified using a SMARTer Ultra Low Input RNA Kit for Sequencing (Clontech, Mountain View, CA, USA). RNA-seq libraries were prepared according to the manufacturer’s protocol (Illumina, San Diego, CA, USA). Libraries were single-endsequenced on a GAIIx sequencer (Illumina, San Diego, CA, USA). Reads were aligned to the mm10 mouse genome using STAR. Aligned read files were analyzed using HOMER (http://homer.ucsd.edu/homer/) to calculate the reads per kb per million mapped reads (RPKM) of RefSeq genes^49^. Expression levels were compared with genes whose average RPKM values over 100, and GO analysis was performed using DAVID Bioinformatics Resources 6.8 and GeneSpring software (Agilent Technologies, Palo Alto, CA, USA).

### Cell culture

Isolated HSCs were seeded on collagen-coated plates in Dulbecco’s modified Eagle’s medium (DMEM) containing 10% fetal bovine serum (FBS) (Biowest, Nuaille, France). After overnight incubation, cells were treated with recombinant TGFβ (10 ng/ml), lipopolysaccharide (10 ng/ml) and bovine serum albumin-conjugated fatty acids: palmitate (100–500 μM), laurate (200 μM), oleate (200 μM) for 24 hours. Human hepatic stellate cell line LX2 were purchased from Merck Millipore (Darmstadt, Germany) and human hepatocellular carcinoma cell line HepG2 were purchased from RIKEN BioResource Center (Ibaraki, Japan). Both cell lines were treated with recombinant human FGF9 protein (1 or 10 ng/mL, R& D Systems, Minneapolis, MN, USA) for 24 hours after serum deprivation.

### Immunocytochemistry of primary HSCs

Primary HSCs were seeded on chambered coverglasses (Nunc LAB-TEK chamber slide system, ThermoFischer Scientific, Waltham, MA, USA) and stimulated as indicated. Cells were fixed with 4% parafolmaldehyde and treated with 0.2% Triton X-100. After blocking with 5% goat serum, cells were stained anti-FGF9 antibody (ab71395, Abcam).

### Construction of FGF9-overexpressed LX2

The entire coding sequence of human FGF9 was amplified by FGF9 Human Tagged ORF Clone (RC210242, Origene, Rockville, MD, USA) using the primer pair set below; 5′-CACGCTACCGGTCTCGAGGCCGCCGCGATCGCCA-3′ (forward) and 5′-GCTCGACCTGCAGGATCCCTAACTTTGGCTTAGAATA-3′ (reverse). FGF9- and GFP (control)-overexpressed LX2 cell lines were produced using lentiviral vector as previously described (FGF9- and control-LX2, respectively)^18^. The FGF9 concentration of the supernatants of these cells was measured by FGF9 Human ELISA Kit (RayBiotech, Norcross, GA, USA) according to the manufacture’s protocol.

### RNA **extraction** and quantitative Real-Time PCR

Total RNA was extracted from the livers or cultured cells using Sepasol reagent or PureLink RNA Micro Kit (Invitrogen, Carlsbad, CA, USA). Quantitative real-time PCR was performed with the StepOnePlus Real-time PCR System using the Fast SYBR Green Master Mix Reagent (Applied Biosystems, Foster City, CA, USA) as previously described^19^. Primers used in this study are listed in Table S2. Data were normalized to 36B4 (mouse) or GAPDH (human) levels and analyzed using the comparative CT method.

### Western blotting analysis

The liver was lysed in tissue lysis buffer (2% SDS, 4M urea, 1 mM EDTA, 150 mM NaCl, 50 mM Tris pH 8.0) supplemented with Protease Inhibitor Cocktail (Merck, Darmstadt, Germany). Proteins were separated by SDS-PAGE and immunoblotted with anti-FGF9 antibody (diluted 1:1000, ab206408, Abcam, Cambridge, UK) and anti-α-Tubulin antibody (diluted 1:1000, #2144, Cell Signaling Technology, Danvers, MA, USA). Immunoblots were detected and analyzed with ECL Prime Western Blotting Detection Reagent and ImageQuant LAS 4000 mini (GE Healthcare, Little Chalfont, UK).

### **siRNA** transfection

Human FGF9-targeting siRNA and non-targeting siRNA were purchased from GE Healthcare Dharmacon (M-011660-01-0005 and D-001206-13-05, Lafayette, CO, USA). siRNAs were transfected to Control-LX2 and FGF9-LX2 according to the manufacture’s protocol. The transfection efficiency was determined by measurement of mRNA expression levels and secreted protein levels in culture media.

### Cell proliferation assay

LX2 or HepG2 (4 × 10^3^ cells) was seeded in 96-well microplates, and treated with recombinant human FGF9 (10 ng/mL). After 96-hour incubation, Cell Proliferation Reagent WST-1 solution (Merck, Darmstadt, Germany) was added, and the plates were incubated at 37 °C for 4 hour. The plates were analyzed using a Microplate Reader (Bio-Rad, Barkeley, CA, USA) at 450 nm with the reference wavelength set at 595 nm.

### Cell migration assay

Transwell cell migration assay was performed using BioCoat Control Inserts with 8.0 µm PET Membrane (Corning, New York, NY, USA). LX2 (1 × 10^4^ cells) or HepG2 (2.5 × 10^4^ cells) were seeded onto the control insert in serum-free medium with or without recombinant FGF9 at a dose of 1 or 10 ng/ml. Cells were incubated with medium containing 2% FBS in the lower chamber for 24 hours. After removal of non-migrating cells on the upper surface of the membrane using cotton swabs, the cells migrated into the lower surface of the membrane were stained with Diff-Quik (Sysmex, Hyogo, Japan) and counted using a microscope in nine visual fields of each sample.

### Apoptosis assay

Caspase-3/7 activity after the induction of apoptosis was measured using Caspase-Glo 3/7 assay (Promega, Madison, WI, USA) as previously described^50^. Cell apoptosis was induced by serum starvation for 48 hours in LX2 cells or anti-Fas antibody (100 ng/mL, SY-001, MBL, Aichi, Japan) for 24 hours in HepG2 cells.

### *In vivo* studies with a subcutaneous tumor xenograft model

HepG2 (2 × 10^5^ cells) and control-LX2 or FGF9-LX2 (1 × 10^6^ cells) were mixed with Matrigel (BD Biosciences, San Jose, CA, USA) and subcutaneously injected into each flank of NOD/ShiJic-*scid*Jcl mice. The length and width of the xenograft tumors were measured once a week, and the tumor volume was calculated as 0.5 × (major axis) × (minor axis)^2^.

### Statistics

Data are presented as mean ± SEM, and *P* < 0.05 was considered statistically significant. Statistical analysis was performed using analysis of variance followed by Tukey-Kramer test. Two-tailed unpaired Student’s *t* test was used to compare two groups.

### Study approval

All animal experiments were conducted in accordance with the guidelines for the care and use of laboratory animals of Tokyo Medical and Dental University. The protocols were approved by the Institutional Animal Care and Use Committee of Tokyo Medical and Dental University (No. 2015-008C, G2018-078A, A2017-111A, A2018-158A and A2019-172A).

## Supplementary information

Supplementary information

## Data Availability

The RNA-seq datasets generated during the current study are available in Gene Expression Omnibus (GEO) of NCBI with the accession number GSE134512 (https://www.ncbi.nlm.nih.gov/geo/query/acc.cgi?acc=GSE134512, token: qrgzacaerhcptqp).
