# Supplementary material for: Upregulation of cancer-associated gene expression in activated fibroblasts in a mouse model of non-alcoholic steatohepatitis
Source: Sci Rep. 2019 Dec 20;9:19601. doi: 10.1038/s41598-019-56039-0 (PMC6925281; doi:10.1038/s41598-019-56039-0)
Supplement: Supplementary file 1 — Supplementary information [file 41598_2019_56039_MOESM1_ESM.pdf]

Supplementary Information

Upregulation of cancer-associated gene expression in activated fibroblasts in a mouse model of non-alcoholic steatohepatitis

Masahiro Asakawa[1], Michiko Itoh[2,3,4]*, Takayoshi Suganami[4,5]*, Takeru Sakai[1], Sayaka Kanai[1], Ibuki Shirakawa[2,4], Xunmei Yuan[1], Tomomi Hatayama[6], Shu Shimada[7], Yoshimitsu Akiyama[7], Katsuhito Fujiu[8], Yutaka Inagaki[9], Ichiro Manabe[10], Shoji Yamaoka[11], Tetsuya Yamada[1], Shinji Tanaka[7], Yoshihiro Ogawa[4,6,12,13]*

[1]Department of Molecular Endocrinology and Metabolism, Graduate School of Medical and Dental Sciences, Tokyo Medical and Dental University, Tokyo, Japan; [2]Department of Organ Network and Metabolism, Graduate School of Medical and Dental Sciences, Tokyo Medical and Dental University, Tokyo, Japan; [3]Kanagawa Institute of Industrial Science and Technology, Kawasaki, Japan; [4]Department of Molecular Medicine and Metabolism, Research Institute of Environmental Medicine, Nagoya University, Nagoya, Japan; [5]Department of Immunometabolism, Nagoya University Graduate School of Medicine, Nagoya, Japan; [6]Department of Medicine and Bioregulatory Science, Graduate School of Medical Sciences, Kyushu University, Fukuoka, Japan; [7]Department of Molecular Oncology, Graduate School of Medical and Dental Sciences, Tokyo Medical and Dental University, Tokyo, Japan; [8]Department of Advanced Cardiology, Graduate School of Medicine, The University of Tokyo, Tokyo, Japan; [9]Center for Matrix Biology and Medicine, Graduate School of Medicine, Tokai University, Isehara, Japan; [10]Department of Disease Biology and Molecular Medicine, Chiba University Graduate School of Medicine, Chiba, Japan; [11]Department of Molecular Virology, Tokyo Medical and Dental University, Tokyo, Japan; [12]Department of Molecular and Cellular Metabolism, Graduate School of Medical and Dental Sciences, Tokyo Medical and Dental University, Tokyo, Japan; [13]Japan Agency for Medical Research and Development, CREST, Tokyo, Japan.

***Corresponding Authors**

Michiko Itoh, Tokyo Medical and Dental University, 1-5-45 Yushima, Bunkyo-ku, Tokyo 113-8510 Japan. Phone: +81-3-5803-4933; E-mail: mito.mem@tmd.ac.jp

Takayoshi Suganami, Nagoya University, Furo-cho, Chikusa-ku, Nagoya 464-8601 Japan. Phone: +81-52-789-3881; E-mail: suganami@riem.nagoya-u.ac.jp

Yoshihiro Ogawa, Kyushu University, 3-1-1, Maidashi, Higashi-ku, Fukuoka 812-8582 Japan. Phone: +81- 92-642-5275; E-mail: yogawa@intmed3.med.kyushu-u.ac.jp

Supplementary Table S1. Gene list upregulated in NASH-fib compared with HSCs and CCl_4-fib.

Accession No.	Gene name	Log2 fold change (NASH-fib vs HSC)	Log2 fold change (NASH-fib vs CCl_4-fib)
NM_147093	Olfr558	4.74	1.38
NM_009814	Casq2	4.67	1.80
NM_001290711	Ebf1	4.42	1.96
NM_010809	Mmp3	4.37	2.47
NM_053106	Lmod1	4.23	1.00
NM_009675	Aoc3	4.22	1.10
NM_026142	3632451O06Rik	4.01	1.51
NM_008318	Ibsp	3.91	4.05
NM_009610	Actg2	3.89	1.02
NM_026056	Cap2	3.89	1.39
NM_010095	Ebf2	3.87	1.63
NM_130866	Olfr78	3.86	1.56
NM_178681	Dgkb	3.86	2.62
NM_144854	Map3k7cl	3.81	2.48
NM_001313753	Map7d2	3.75	1.89
NM_177906	Opcml	3.66	2.91
NM_080846	Higd1b	3.60	1.34
NM_010934	Npy1r	3.48	2.26
NM_145136	Myocd	3.43	1.37
NM_001002927	Penk	3.42	1.53
NM_013518	Fgf9	3.37	2.83
NM_013703	Vldlr	3.36	1.02
NM_011918	Ldb3	3.35	1.05
NM_010944	Musk	3.30	2.33
NM_177074	Slc38a11	3.29	2.45
NM_009135	Scn7a	3.27	1.80
NM_001081432	Ptprq	3.25	2.69
NM_023129	Pln	3.17	1.36
NM_001310677	Bmp3	3.11	4.78
NM_001122756	Corin	3.09	1.43
NM_031169	Kcnmb1	3.09	1.52
NM_010818	Cd200	3.04	1.59
NM_001286653	Rcan2	3.02	1.71
NM_053191	Pi15	2.99	2.73
NM_016711	Tmod2	2.94	1.69
NM_146010	Tspan8	2.94	1.73
NM_001290435	Gabrq	2.92	2.04
NM_028903	Scara5	2.89	1.42
NM_018881	Fmo2	2.85	2.18
NM_008365	Il18r1	2.84	1.45
NM_001309233	Lrrc31	2.84	4.05
NM_001114385	Chrdl1	2.76	3.84
NM_138674	Pkhd1l1	2.75	1.08
NM_001204202	Spp1	2.73	2.08
NM_011311	S100a4	2.71	1.56

NM_001243837	C7	2.67	2.18
NM_080467	Atp6v0a4	2.65	2.08
NM_013415	Atp1b2	2.64	1.03
NM_028182	Sh2d4a	2.64	1.63
NM_008030	Fmo3	2.63	4.27
NM_001285422	Bdnf	2.59	2.91
NM_138650	Dgkg	2.57	1.66
NM_001253754	Gpm6a	2.57	1.29
NM_029639	Plet1	2.57	1.93
NR_045702	AW549542	2.56	1.63
NM_001301375	Fst	2.55	1.93
NM_001199556	AW551984	2.48	1.18
NM_008485	Lamc2	2.47	1.40
NM_010171	F3	2.41	1.43
NM_001081088	Lrp2	2.38	1.61
NM_011330	Ccl11	2.35	1.34
NM_001198565	Sulf1	2.34	1.22
NM_021050	Cftr	2.33	3.22
NM_144556	Lgi4	2.30	1.64
NM_008557	Fxyd3	2.30	1.73
NM_013657	Sema3c	2.29	2.26
NM_023887	Gcnt2	2.29	1.14
NM_009250	Serpini1	2.29	1.70
NM_001146299	Sh3rf2	2.28	2.55
NM_010867	Myom1	2.28	1.74
NM_080451	Synpo2	2.27	1.47
NM_023755	Tfcp2l1	2.26	1.20
NM_001164585	Dnajc6	2.25	1.52
NM_021355	Fmod	2.23	1.07
NM_009549	Zfp185	2.20	1.77
NM_009763	Bst1	2.20	2.68
NM_008716	Notch3	2.18	1.52
NM_025557	Pcp4l1	2.18	1.63
NM_145492	Zfp521	2.15	1.92
NM_001145937	Tenm3	2.13	1.76
NM_007562	Bnc1	2.10	1.37
NM_009154	Sema5a	2.09	1.59
NM_008008	Fgf7	2.06	1.78
NM_027519	Medag	2.03	1.31
NM_010265	Gcnt1	2.03	2.25
NM_001205219	Sorbs2	2.01	1.10
NM_173447	Ephb1	2.00	1.22
NM_001255997	Cacna1c	1.98	2.09
NM_007426	Angpt2	1.94	2.15
NM_008013	Fgl2	1.91	2.15
NM_008607	Mmp13	1.90	6.31
NM_212435	Foxp2	1.89	1.87
NM_022879	Myl7	1.87	1.38
NM_010795	Mgat3	1.86	1.03

NM_201600	Myo5b	1.84	1.32
NM_008905	Ppfibp2	1.83	1.92
NM_011704	Vnn1	1.83	1.95
NM_011454	Serpinb6b	1.80	1.61
NM_018760	Slc4a4	1.76	1.35
NM_009196	Slc16a1	1.75	1.54
NM_007735	Col4a4	1.72	2.44
NM_011448	Sox9	1.70	1.16
NM_029102	Glt8d2	1.70	2.15
NM_010518	Igfbp5	1.70	1.70
NM_001111110	Cmah	1.69	2.17
NM_001099217	Ly6c2	1.67	1.57
NM_008760	Ogn	1.66	1.73
NM_007734	Col4a3	1.66	2.31
NM_145539	Tm4sf4	1.62	3.59
NM_001033220	AU021092	1.59	1.68
NM_001297596	Inpp4b	1.59	3.13
NM_201529	Lmo7	1.58	1.59
NM_011169	Prlr	1.57	2.58
NM_172496	Cobl	1.52	1.28
NM_026535	Serpina12	1.49	2.95
NM_012050	Omd	1.47	3.40
NM_198967	Tmtc1	1.45	1.54
NM_175460	Nmnat2	1.45	2.24
NM_011171	Procr	1.43	1.09
NM_001271599	Sort1	1.41	1.49
NM_001081243	Filip1	1.39	1.40
NM_011048	Pcsk6	1.38	2.22
NM_001163640	Chn2	1.37	2.43
NM_008605	Mmp12	1.36	1.57
NM_147218	Abca6	1.35	1.30
NM_018732	Scn3a	1.31	2.86
NM_011211	Ptprd	1.30	1.40
NM_153145	Abca8a	1.29	1.55
NM_172992	Phtf2	1.29	1.15
NM_016675	Cldn2	1.28	5.21
NM_001159383	Gjc1	1.27	1.21
NM_026821	Lurap1l	1.27	2.54
NM_001310439	Adam22	1.26	1.74
NM_009014	Rad51b	1.23	1.66
NM_001190374	Adamtsl3	1.23	1.99
NM_013904	Hey2	1.21	1.17
NM_177025	Cobll1	1.20	1.32
NM_053108	Glrx	1.20	1.97
NM_029879	Rgs7bp	1.19	1.82
NM_010831	Sik1	1.18	1.18
NM_008862	Pkia	1.18	1.12
NM_001025559	Sox6	1.18	1.36
NM_001135149	Slc39a8	1.18	2.30

NM_176837	Arhgap18	1.16	1.97
NM_001170537	Mef2c	1.15	1.66
NM_027924	Pdgfd	1.14	1.39
NM_206958	Ltbp1	1.12	1.54
NM_001163645	Osbpl3	1.12	1.72
NM_026346	Fbxo32	1.11	1.25
NM_009848	Entpd1	1.10	2.13
NM_198111	Akap6	1.10	1.61
NM_016719	Grb14	1.08	1.66
NM_018761	Ctnnal1	1.08	1.05
NM_001130513	Ace2	1.07	2.19
NM_019397	Egfl6	1.06	1.02
NM_201411	Flrt1	1.06	1.88
NR_003966	Atp10d	1.06	1.53
NM_025626	Fam107b	1.06	1.66
NM_022032	Perp	1.05	1.56
NM_021342	Kcne4	1.04	1.53
NM_001012324	Ecm2	1.03	2.07
NM_013598	Kitl	1.03	1.42
NM_172563	Hlf	1.02	1.19
NM_010576	Itga4	1.02	2.78

Supplementary Table S2. Primers used in this study.

Gene name	Primer	Sequence	Product size (bp)
36B4 (mouse)	forward	5'-GGCCCTGCACTCTCGCTTTC-3'	124
	reverse	5'-TGCCAGGACGCGCTTGT-3'	
GAPDH (human)	forward	5'-TGGGTGTGAACCATGAGAAG-3'	76
	reverse	5'-GCTAAGCAGTTGGTGGTGC-3'	
Fgf9 (mouse)	forward	5'-ACTCTACCTCGGCATGAACGA-3'	145
	reverse	5'-TCTCCTTCCGGTGTCCACAT-3'	
FGF9 (human)	forward	5'-GAAAGACCACAGCCGATTTG-3'	99
	reverse	5'-TTCATCCCGAGGTAGAGTCC-3'	
IL1A (human)	forward	5'-TGGTAGTAGCAACCAACGGGA-3'	215
	reverse	5'-ACTTTGATTGAGGGCGTCATTC-3'	
IL1B (human)	forward	5'-TCCTGCGTGTTGAAAGATGATAA-3'	67
	reverse	5'-TTGGGTAATTTTTGGGATCTACACT-3'	
CCL2 (human)	forward	5'-GATCTCAGTGCAGAGGCTCG-3'	153
	reverse	5'-TGCTTGTCCAGGTGGTCCAT-3'	
CXCL8 (human)	forward	5'-TTTTGCCAAGGAGTGCTAAAGA-3'	194
	reverse	5'-AACCCTCTGCACCCAGTTTTC-3'	
COL1A1 (human)	forward	5'-GGTCAGATGGGCCCCCG-3'	86
	reverse	5'-GCACCATCATTTCCACGAGC-3'	
TGFB1 (human)	forward	5'-CGACTCGCCAGAGTGGTTAT-3'	86
	reverse	5'-GGCGAAAGCCCTCAATTTCC-3'	

Supplementary Figures

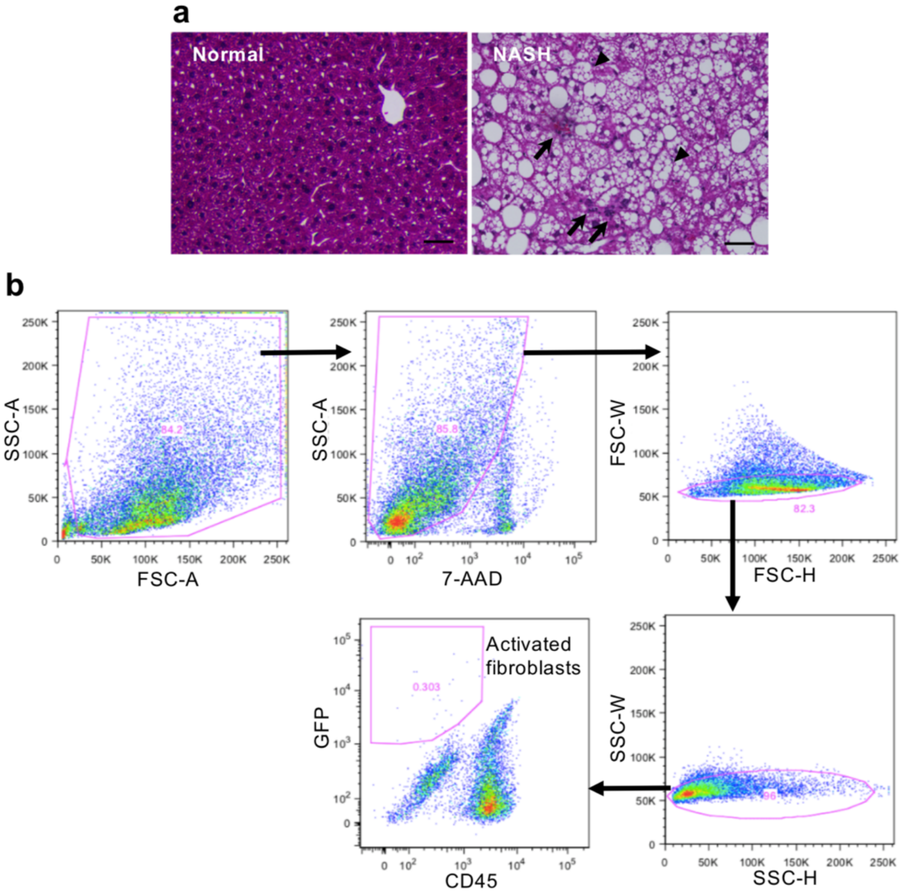

Supplementary Figure S1. Histological analysis of the livers from MC4R-KO mice crossed with Col1a2-GFP transgenic mice fed WD for 20 weeks.

(a) Representative images of HE staining of the livers. Normal, wild-type (WT) mice fed standard diet (SD); NASH, Col1a2-GFP Tg MC4R-KO mice fed Western diet (WD) for 20 weeks. Arrows, inflammatory cell infiltration; arrowheads, ballooning degeneration. Scale bars, 50 µm. (b) Gating strategy for identification of GFP-positive activated fibroblasts in non-parenchymal cells determined by flowcytometry.

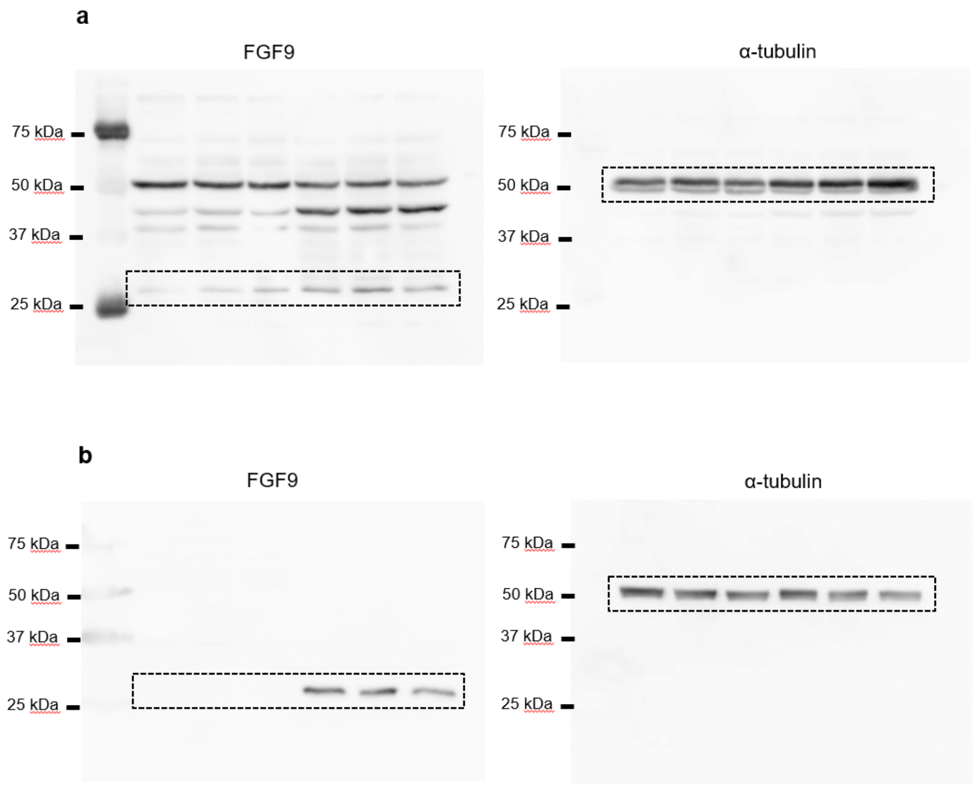

Supplementary Figure S2. Uncropped Western blots.

Uncropped Western blots for Fig. 3b (a), and Fig. 4b (b). Boxes indicate cropped area.

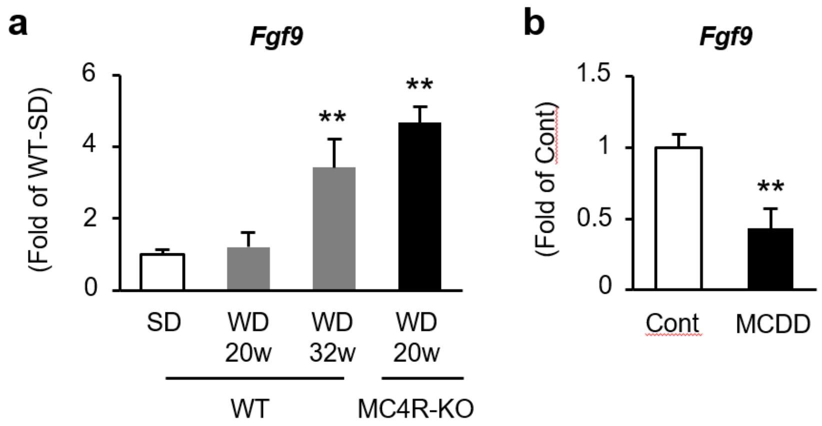

Supplementary Figure S3. *Fgf9* mRNA expression in livers from different NASH models.

(a) mRNA expression levels in livers from wild-type and MC4R-KO mice fed WD for indicated periods. ** $P < 0.01$ vs. WT-SD. $n = 6$. (b) mRNA expression levels in methionine and choline–deficient diet (MCDD) model. Cont, wild-type mice fed control diet; MCDD, wile-type mice fed MCDD for 8 weeks. ** $P < 0.01$ vs. Cont. $n = 6$.

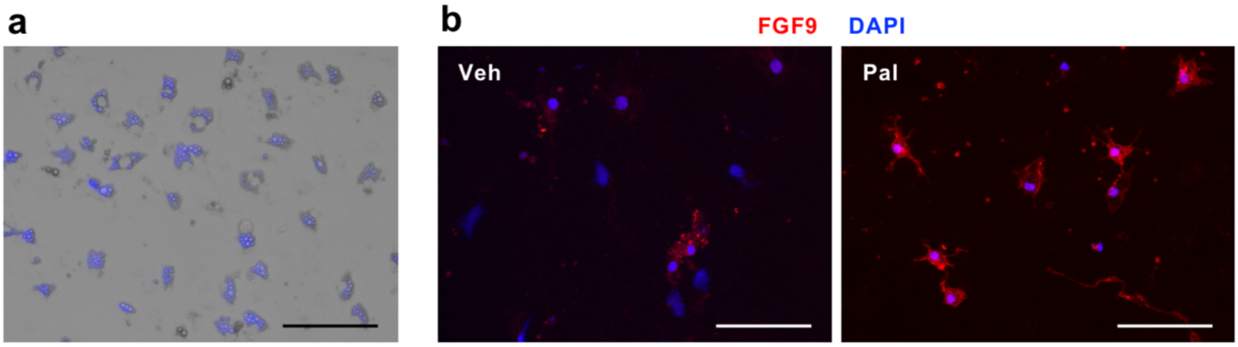

Supplementary Figure S4. FGF9 immunocytochemistry of primary HSCs treated with palmitate.

(a) The representative image of isolated HSCs before palmitate treatment showing the autofluorescence excited by UV light merged with bright field. (b) Primary HSCs were treated with palmitate (500 μM) for 24 hours, and stained with FGF9 antibody. FGF9 immunocytochemistry was performed twice, and the representative images were indicated. Veh, vehicle; Pal, palmitate. Scale bars, 100 μm.

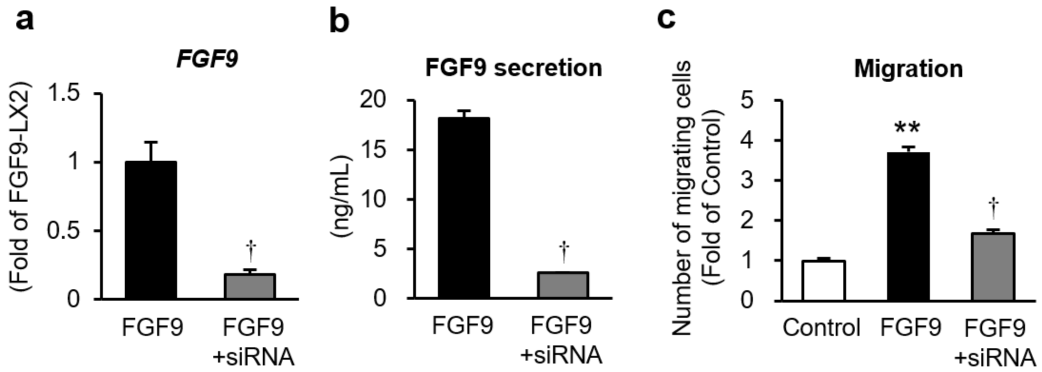

Supplementary Figure S5. FGF9 enhances cell migration in stably FGF9-overexpressed LX2 cells.

Knockdown efficiency by siRNA in FGF9-LX2 evaluated by *FGF9* mRNA expression levels (a) and FGF9 secretion into culture supernatants (b). (c) Migration activity of FGF9-LX2 cells and the effect of FGF9 knockdown. ** $P < 0.01$ vs. control-LX2. † $P < 0.01$ vs. FGF9-LX2. $n = 4$. Data represent mean ± SEM.

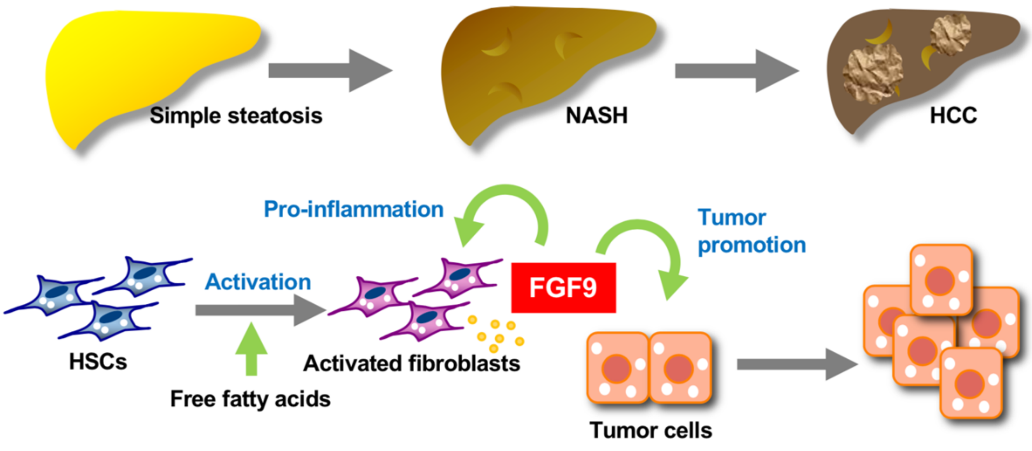

Supplementary Figure S6. Graphical summary.

NASH, non-alcoholic steatohepatitis; HCC, hepatocellular carcinoma; HSCs, hepatic stellate cells; FGF9, fibroblast growth factor 9.
